# Ultrasound-Guided Interpectoral and Pectoserratus Plane Blocks in Breast Surgery: An Alternative Option to General Anaesthesia in an Elderly Woman with a Complex Medical History

**DOI:** 10.3390/life12122080

**Published:** 2022-12-11

**Authors:** Carmelo Pirri, Debora Emanuela Torre, Astrid Ursula Behr, Raffaele De Caro, Carla Stecco

**Affiliations:** 1 Department of Neurosciences, Institute of Human Anatomy, University of Padova, 35121 Padova, Italy; 2 Department of Cardiac Anesthesia and Intensive Care Unit, Cardiac Surgery, Ospedale dell’Angelo, 30174 Venice Mestre, Italy; 3 Operative Unit of Anesthesia and Resuscitation, Hospital of Camposampiero, 35012 Camposampiero, Italy

**Keywords:** regional anaesthesia, PECS II block, opioids sparing, fascia, fascial block, age, dementia, ultrasound

## Abstract

With an incidence of over 1.5 million worldwide per annum, breast cancer continues to be the most common cancer affecting the female population. The main and most effective treatment in over 40% of these patients is a primary neoplasm resection. General anaesthesia, at times in association with loco-regional anaesthetics, is the most commonly used anaesthesia technique for radical mastectomies. Nausea, vomiting, and considerable postoperative pain, which are commonly experienced side effects and complications of general anaesthesia, tend, however, to augment most patients’ post-surgical morbidity. A growing body of research has shown that loco-regional anaesthesia often used together with and, in some cases, in the substitution of general anaesthesia can be a safe, effective alternative. This work is a case report regarding a 94-year-old elderly patient who was anaesthetised during a left radical mastectomy using exclusively combined interpectoral and pectoserratus plane blocks.

## 1. Introduction

With an incidence of over 1.5 million worldwide per annum, breast cancer continues to be the most common cancer affecting the female population [1,2]. The prime and most effective treatment in most cases is surgery, and a neoplasm resection is in fact the primary treatment option in over 40% of patients [3]. Moreover, while it is true that breast-conserving surgery (BSC) and sentinel lymph node biopsy are widely used, radical mastectomy is the standard surgical treatment for female patients who have contraindications for BSC or who have a positive sentinel lymph node biopsy [4]. Although general anaesthesia (GA), often in association with loco-regional procedures, is the most commonly used anaesthetic technique for radical mastectomy surgery, its complications and side effects, such as nausea, vomiting, and considerable postoperative pain, increase postoperative morbidity [5]. A growing body of research has been dedicated to investigating the use of loco-regional anaesthesia either in association with or, in some cases, in substitution of GA during breast surgery [6,7]. Those studies have shown that these techniques can reduce postoperative complications and provide the optimal control of postoperative pain, thus minimising the need for the use of opioids.

The regional anaesthetic techniques most commonly used for breast surgery are: a thoracic epidural block, thoracic paravertebral block, erector spinae plane block, deep and superficial serratus anterior blocks, as well as interpectoral plane (IPP) and pectoserratus plane (PSP) blocks [7]. The high risk of pneumothorax, haemorrhage, dural penetration, and hypotension linked to these techniques needs, nevertheless, to be taken into consideration [8]. First described by Blanco et al. in 2012, the Pectoralis II (PECS II) fascial block is an inter-fascial plane block between the pectoralis major and minor muscles and between the pectoralis minor and serratus anterior muscles at the levels of the third and fourth ribs along the mid-axillary line. The low incidence of complications seemingly connected to the novel approach to breast cancer surgery is at least in part due to ultrasound imaging guidance [9]. Moreover, a recent international consensus study involving experts using a three-round Delphi method produced a standardised nomenclature of regional anaesthetic techniques for the abdominal wall, paraspinal, and chest wall [10]. Based on the consensus study’s recommendations, the original PECS II block should now be referred to as a combination of IPP and PSP [10].

The current work describes the case of an elderly patient who underwent a left radical mastectomy due to breast cancer and a positive sentinel lymph node biopsy under ultrasound-guided IPP and PSP blocks anaesthesia.

## 2. Case Report

**Figure 1:**
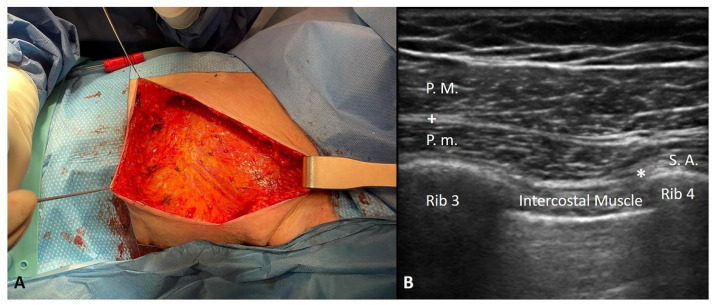
(**A**) A left radical mastectomy due to breast cancer and positive sentinel lymph node biopsy in an elderly female patient. (**B**) Combined interpectoral and pectoserratus plane blocks: a 50 mm Pajunk echogenic needle was used to inject ropivacaine 0.5% 10 mL (+: in the inter-fascial plane between the major and minor pectoralis muscles) + 20 mL (*: in the inter-fascial plane between the pectoralis minor and serratus anterior muscles).

A 94-year-old woman with breast cancer and a positive sentinel lymph node biopsy was scheduled for a left radical mastectomy (Figure 1A). The patient’s medical history included pathologies such as dementia, hypertension, Mobitz I type BAV, hypothyroidism (the patient was receiving replacement therapy), osteoporosis, megaloblastic anaemia, recent interstitial pneumonia, and METS > 4 < 10. According to the classification of the American Society of Anesthesiologists (ASA), she was an ASA III, thus a patient with a severe systemic disease that was not life threatening.

The surgical team decided against using GA, orotracheal intubation, and intra or postoperative opioids because of the complications (delirium, disrupted psychomotor performance, cardio-respiratory depression, and ileus) linked to their use, and they hoped to discharge her as soon as possible after surgery in view of her age (94 years), clinical complexity, and comorbidities. The team opted for performing combined IPP and PSP blocks in the recovery room. This was carried out using an ultrasound linear probe (high frequency) and an ultrasound Sonosite Edge II machine in the following manner: a 50 mm Pajunk echogenic needle was used to inject ropivacaine 0.5% 10 mL (in the inter-fascial plane between the major and minor pectoralis muscles) +20 mL (in the inter-fascial plane between the pectoralis minor and serratus anterior muscles) (Figure 1B). 

The patient was transferred to the operating room 20 min after the block was performed; at the time the incision was carried out, the Numerical Rating Scale (NRS) was equal to 0. As the patient seemed uncomfortable in the unfamiliar environment, a low dose propofol infusion (1.4 mg/kg/h) was begun.

The patient’s Richmond Agitation-Sedation Scale (RASS) was equal to -1. Her ECG, non-invasive blood pressure (NIBP), oxygen saturation (SpO_2_), end-tidal CO_2_ (EtCO_2_), and Bispectral Index (BIS) were all being monitored.

The patient’s BIS was between 80 and 65; moreover, she maintained spontaneous breathing with a nasal cannula O_2_ 3 lt/min, maintaining a saturation between 94 and 98% (saturation in ambient air 93%) and had no episodes of apnoea. The patient was haemodynamically stable throughout the surgery. There was a single hypertensive peak of 150/90 mmHg thirty minutes after the incision was made; it was treated with 10 mg of urapidil. During surgery, 70 mg of ketamine was administered in boluses (30 mg pre-incision, 20 mg 10 min post-incision, and 20 mg 30 min post-incision). Before suturing and inserting the drainage, the surgeon infiltrated the surgical wound with 200 mg of lidocaine. Throughout the procedure, the patient’s numeric rating scale (NRS) was between 0 and 3 (required for awake patients). At the end of the surgery, the patient was transferred to the recovery room; her vital parameters were stable and pain control was good. An hour later, she was transferred to the ward with an NRS = 0.

During the post-operative period, a 1 g paracetamol solution for infusion was administered 3 h after surgery (1 h after she arrived in the ward), 8 h later, and 16 h after that. The patient did not require rescue analgesia, and she was discharged from hospital 24 h after the surgery.

## 3. Discussion

In the case described above, analgesia of a complex elderly patient’s pectoral, mammary, inframammary and axillary regions during a mastectomy was provided by IPP and PSP blocks. The fourth, fifth, and sixth intercostal nerves supply the mammary region, whereas the medial (C8-T1) and lateral (C5-C7) pectoral nerves innervate the pectoralis major and minor muscles and the overlying fasciae [9]. 

The local anaesthetic spread between the clavipectoral fascia and the superficial border of the serratus anterior muscles anaesthetising the anterior cutaneous branches of the intercostal nerves situated between the thoracic spinal nerve (T4-6), the intercostobrachial and the long thoracic nerve [10]. To reduce the risk of a vascular and pleural puncture during the procedure, the direction in which the needle was inserted was medial to lateral. In addition to its relative simplicity, the procedure described carries a minimal risk of injuries to the pleura and blood vessels and more reliably provides analgesia with respect to the infiltration of the surgical wound with a local anaesthetic [1]. Indeed, Torre et al. reported that PECS II could potentially provide analgesia for lateral incisions involving minimally invasive cardiac surgery [11]. 

At the current time, IPP and PSP blocks are commonly used as part of multimodal analgesia for breast surgery, for which they were originally developed. Several studies have demonstrated that they are effective in reducing nausea, vomiting, postoperative narcotic consumption, and the length of the hospital stay [12]. In the case described here, ultrasound-guided IPP and PSP blocks were used to avoid GA in an elderly, complicated patient. This was done to circumvent the side effects and complications of GA which, of course, include nausea, vomiting, considerable postoperative pain, and increased morbidity in many breast surgery patients and, in particular, in elderly, complex ones [4]. It goes without saying that the surgical team wanted to avoid, if possible, an orotracheal intubation as well as intra and post-operative opioids in view of their complications (delirium, worse psychomotor performance, cardio-respiratory depression, and ileus). At the same time, they sought to discharge the patient as soon as possible to avoid their disorientation in an unfamiliar place and aggravating her comorbidities. 

As a final note, we would like to point out that the fibrous component of the fascial tissues increases with aging [13,14]; this could explain their capacity to contain an anaesthetic for a longer time, thus prolonging its effect. In other words, the analgesic effect may last longer because the fascial tissues of an elderly person absorb and spread those drugs in a different manner, with respect to younger individuals. 

## 4. Conclusions

In conclusion, ultrasound-guided IPP and PSP blocks were found to be a simple, safe, and effective technique to anaesthetise a difficult, elderly patient undergoing a mastectomy. Further studies will be able to confirm its safety and efficacy in patients of all ages with a complex medical history and confirm that it has no or minimal complications and side effects.

## Data Availability

The data presented in this study are available on request from the corresponding author. The data are not publicly available due to privacy.
